# Factors promoting health-related quality of life in people with rheumatic diseases: a 12 month longitudinal study

**DOI:** 10.1186/1471-2474-12-102

**Published:** 2011-05-20

**Authors:** Susann Arvidsson, Barbro Arvidsson, Bengt Fridlund, Stefan Bergman

**Affiliations:** 1 Research and Development Centre Spenshult, Spenshult hospital for rheumatic diseases, SE-313 92 Oskarström, Sweden; 2 School of Health Sciences, Jönköping University, Jönköping, Sweden; 3 School of Social and Health Sciences, Halmstad University, Halmstad, Sweden; 4 Gjøvik University College, Faculty of Nursing Science, Gjøvik, Norway

## Abstract

**Background:**

Rheumatic diseases have a significant adverse impact on the individual from physical, mental and social aspects, resulting in a low health-related quality of life (HRQL). There is a lack of longitudinal studies on HRQL in people with rheumatic diseases that focus on factors promoting HRQL instead of risk factors. The aim of this study was to investigate the associations between suggested health promoting factors at baseline and outcome in HRQL at a 12 month follow-up in people with rheumatic diseases.

**Methods:**

A longitudinal cohort study was conducted in 185 individuals with rheumatic diseases with questionnaires one week and 12 months after rehabilitation in a Swedish rheumatology clinic. HRQL was assessed by SF-36 together with suggested health factors. The associations between SF-36 subscales and the health factors were analysed by multivariable logistic regressions.

**Results:**

Factors predicting better outcome in HRQL in one or several SF-36 subscales were being younger or middle-aged, feeling painless, having good sleep structure, feeling rested after sleep, performing low effort of exercise more than twice per week, having strong sense of coherence (SOC), emotional support and practical assistance, higher educational level and work capacity. The most important factors were having strong SOC, feeling rested after sleep, having work capacity, being younger or middle-aged, and having good sleep structure.

**Conclusions:**

This study identified several factors that promoted a good outcome in HRQL to people with rheumatic diseases. These health factors could be important to address in clinical work with rheumatic diseases in order to optimise treatment strategies.

## Background

Rheumatic diseases have a significant adverse impact on the individual from physical, mental and social aspects [1], resulting in a low health-related quality of life (HRQL) [2-4]. Recent research suggests that individuals with rheumatoid arthritis (RA) who receive a multi-disciplinary team-based care at a rheumatology clinic get improved HRQL and also a decrease in symptoms from the joints and in inflammatory parameters up to 12 months after an intervention [5,6]. However, little attention has been paid on studying the effect of health factors (salutogenesis) instead of risk factors (pathogenesis) within the context of rheumatic care.

The objective of a salutogenetic approach is to enhance the individual's resources to become more resistant to the debilitating effects of the disease. The focus is on the human strengths and factors that create the conditions for health. If an individual experiences life as *understandable *and *manageable *as well as finds it *meaningful *in dealing with problems that arise, then this individual has a greater ability to stay healthy and to have a strong sense of coherence (SOC). The most important determinant for SOC is personal relationships and not the social environment [9,10]. Women with fibromyalgia who had a stronger SOC perceived greater well-being, and felt more hopeful, free, valuable and more like others [11]. Older individuals with RA who have social support reported better self-care behaviour [12] and individuals with systemic lupus erythematosus (SLE) with a strong SOC had the ability to cope with disease-related stressors and get better HRQL [13]. In people with RA and osteoarthritis, exercise intervention could have a moderate positive effect on physical activity behaviour [14,15] but does not always improve HRQL in people with RA [15]. Several other factors can be supposed to promote health in people with rheumatic diseases.

In order to optimise treatment strategies within the clinical practice it would be valuable to identify health factors that affect HRQL in a positive direction. There is a lack of longitudinal studies on HRQL in people with rheumatic diseases that focus on factors predicting HRQL instead of risk factors.

The aim of this study was to investigate the associations between suggested health promoting factors at baseline and outcome in HRQL at a 12 month follow-up in people with rheumatic diseases.

## Methods

### Design and setting

The study was designed as a longitudinal cohort study in people with rheumatic diseases, with a questionnaire one week and 12 months after a completed rehabilitation program in a Swedish rheumatology clinic. The individuals, aged 18 years or older and primarily from the middle and south of Sweden, had undergone an inpatient rehabilitation program which has been referred by physicians. Focus during the stay at the clinic was physical training and medical help but also to support, teach and provide individual advice for self-care.

#### Participants and dropouts

All individuals (n = 249) with at least one diagnosed rheumatic disease who received three weeks of rehabilitation at the clinic during the period February - June 2007 and with no great difficulties to read and complete the Swedish questionnaire were asked to participate in the study. There were 200 (80%) individuals included one week after rehabilitation and at the 12 month follow-up there were 185 (74%) individuals who responded to the questionnaire.

At the 12 month follow-up there were 15 individuals who decided not to participate, of whom one (7%) was a man and 14 (93%) were women. Their mean age was 62 years (24-81 years). Nine (60%) of the individuals were living alone and six (40%) of the individuals were living with somebody. Seven (47%) had grade school as highest education, five (33%) had secondary school and three (20%) had college/university as highest education. There were six (40%) of the individuals who had an inflammatory joint disease, four (27%) had a systemic rheumatic disease, two (13%) had osteoarthritis, and three (20%) had local/general pain. Additionally, there was a higher percentage in the group of dropouts who were women, were living alone and who had a systemic rheumatic disease compared to the individuals who completed the study (Table 1).

**Table 1:** Socio-demographic and supposed health-factors in a population with rheumatic diseases one week after rehabilitation.

Characteristics	One week		Characteristics	One week	
	**n = 185**	**%**		**n = 185**	**%**

Sex			Social support		
Women	139	75	18-29	38	20
Men	46	25	13-17	50	27
			11-12	40	22
Age (years)			10 Very good	53	29
70-88	42	22	No answer	4	2
61-69	45	24			
52-60	49	27	Alcohol habit		
18-51	49	27	Rarely/never	78	42
			Monthly	50	27
Feeling painless			Weekly	57	31
8-10	21	11			
6-7	57	31	Immigrant status		
4-5	49	27	Immigrant	23	12
0-3 Nearly painless	58	31	Swede	162	88
					
Sleep structure			Civil status		
Big/very big problem	88	48	Living alone	61	33
Moderate problem	60	32	Living with somebody	124	67
No/small problem	37	20			
			Education		
Feeling rested			Grade school	75	41
Big/very big problem	76	41	Secondary school	58	31
Moderate problem	53	29	College/university	52	28
No/small problem	56	30			
			Work capacity		
Diet			0%	64	34
General diet	161	87	25-100%	53	29
Special diet	24	13	Retired	51	28
			No answer	17	9
High effort of exercise					
Irregularly/never	109	59	Socioeconomic status,		
1 time per week	25	13	main occupation		
>2 times per week	51	28	Group A	70	38
			Group B	44	24
Medium effort of exercise			Group C	56	30
Irregularly/never	100	54	Group D	12	6
1 time per week	19	10	No answer	3	2
>2 times per week	66	36			
			Socioeconomic status,		
Low effort of exercise			current occupation		
Irregularly/never	50	27	Group A	26	14
1 time per week	15	8	Group B	12	7
>2 times per week	120	65	Group C	32	17
			Group D	90	49
Perform hobbies			No answer	25	13
Rarely/never	60	32			
Often/sometimes	125	68	Rheumatic disease		
			Local/general pain	25	14
Feeling sexual lust			Osteoarthritis	24	13
Rarely/never	81	44	Systemic rheumatic disease	19	10
Often/sometimes	87	47	Infl. joint disease	117	63
No answer	17	9			
SOC					
21-55	48	26			
56-66	49	27			
67-75	48	26			
75-90 Very good	40	21			

Group A: Manual workers

Group B: Assistant no manual employees

Group C: Intermediate/higher employees and upper-level executives

Group D: Others

### Data collection

A cover letter, an informed consent and a questionnaire were sent to the individuals one week after discharge from the clinic. The informed consent and, in the event that the individuals decided to participate, the completed questionnaire were returned to the first author. Three weeks after the discharge from the clinic a reminder was done by a telephone call. A similar procedure was carried out at 12 months after completed rehabilitation.

#### Instruments

The salutogenetic perspective was the starting point in the selection of measuring instruments and the following areas were chosen: HRQL, feeling painless, sleep structure, feeling rested, diet, exercise habits, performing hobbies, feeling sexual lust, SOC, social support, alcohol habit, immigrant status, civil status, education, work capacity, socioeconomic status - main occupation, socioeconomic status - current occupation, and rheumatic disease.

• In order to assess HRQL the Short Form-36 health survey (SF-36), a general questionnaire was used [16]. The Swedish version of SF-36 has shown good reliability and validity [17-19]. The SF-36 gives eight subscales assessing different aspects of HRQL: Physical Functioning (PF), Role - Physical (RP), Bodily Pain (BP), General Health (GH), Vitality (VT), Social Functioning (SF), Role - Emotional (RE) and Mental Health (MH). The score for each of the eight subscales ranged from 0 to100. A higher score indicated better health [16].

• Feeling painless was assessed by a general question about the average pain intensity the past week and the response ranged from 0 to10, where a lower score indicated less pain (study specific question).

• Sleep structure was assessed by three questions regarding experiences of problems falling asleep, frequent awakenings during the night and early morning awakening. A fourth question, assessing not feeling rested after sleep, represented a more qualitative aspect of non-restorative sleep and was introduced separately in the analyses as feeling rested. Sleep structure and feeling rested were assessed with five alternatives: (1) No problems; (2) Small problems; (3) Some problems; (4) Great problems; and (5) Very great problems [20-22].

• Diet was assessed with six alternatives: (1) General diet; (2) Only lacto-vegetarian diet; (3) Most lacto-vegetarian diet but occasionally eat fish and egg; (4) Vegan diet; (5) Gluten-free diet; and (6) Other diet, please describe [23].

• Exercise habits were assessed by three questions about the frequency of exercise on different levels of effort: high, medium and low effort. There were five alternatives: (1) Never; (2) Irregularly; (3) One time per week; (4) Two times per week; and (5) Three or more times per week [24].

• Performing hobbies was assessed with four alternatives: (1) Never; (2) Rarely; (3) Sometimes; and (4) Often (study specific question).

• Feeling sexual lust was assessed with four alternatives: (1) Never; (2) Rarely; (3) Sometimes; and (4) Often [25].

• The SOC is a questionnaire based on Antonovsky's salutogenic theory and was used to assess the sense of coherence, measured by comprehensibility, manageability and meaningfulness. The version with 13 questions was selected [10] since this shorter version has shown good reliability and validity [26,27]. The score for each of the questions ranged from 1 to 7. A higher score indicated a strong SOC [27].

• The Social Network and Social Support Scale (SNASS) is a questionnaire used to assess social network and social support and consists of 19 items. SNASS has shown good reliability and validity [28,29]. The 10 questions that affect emotional support and practical assistance were included in the present study. The score for each of the 10 questions ranged from: Yes, absolutely = one point; Yes, partly = two points; and No = three points. A lower score indicated a strong emotional support and practical assistance [28,29].

• Alcohol habit assessed the frequency of alcohol use with five alternatives: (1) Never; (2) Very seldom; (3) Monthly; (4) One or two times per week; and (5) Daily [20].

• Work capacity assessed the degree of the work capacity with six alternatives: (1) 100%; (2) 75%; (3) 50%; (4) 25%; (5) No work capacity; and (6) Retired (study specific question).

• Socioeconomic status was based on the occupation and classified according to the Swedish socioeconomic classification system, SEI [30].

• The diagnosed rheumatic disease was obtained from medical records.

A pilot study to test the questionnaire was done on 24 other individuals that varied in sex, age, and occurrence of diseases or not. Thereafter some minor adjustments were made in the layout of the questionnaire and some study specific questions were clarified to reduce the risk of misinterpretation.

### Statistical procedure and analysis

The *SF-36 *outcome scores were dichotomised with regard to the mean values in the population for each of the eight subscales (1 ≥mean and 0 <mean). *Age *and *feeling painless *were divided into quartiles. Moderate or big problems in any of the three questions about sleep were considered as representative for problems with *sleep structure*. In the analyses about *sleep structure *and *feeling rested*, the answers were merged into three groups with scores 1-2 representing no/small problems, score 3 representing moderate problem, and scores 4-5 representing big/very big problems. In the analyses about *diet*, the answers were merged into two groups with score 1 representing general diet and scores 2-6 representing special diet. In the analyses about *exercise *habits, the answers from each of the three questions were merged into three groups with scores 1-2 representing never/irregularly, score 3 representing one time per week and scores 4-5 representing >2 times per week. In the analyses about *performing hobbies *and *feeling sexual lust*, the answers to each question were merged into two groups with scores 1-2 representing never/rarely and scores 3-4 representing sometimes/often. The questions from *SOC *and *social support *were calculated and then the values were divided into quartiles. In the analyses about *alcohol habit*, the answers were merged into three groups with scores 1-2 representing never/rare, score 3 representing monthly, and scores 4-5 representing weekly. In the analyses about *work capacity*, the answers were merged into three groups with scores 1-4 representing 25-100% work capacity, score 5 representing no work capacity, and score 6 representing retired. *Socioeconomic status *was classified according to the Swedish socioeconomic classification system, SEI [30], and the 18 basic socioeconomic classes were merged into four groups: manual workers, assistant no manual employees, intermediate/higher no manual employees including upper level executives, and others. The group "others" included self-employed, farmers, housewives, and students [31]. The *rheumatic diseases *were merged into four groups: inflammatory joint disease, systemic rheumatic disease, osteoarthritis and local/general pain [32].

The statistical package SPSS for Windows, Release 17.0 was used in the analysis. The t-test was used for statistical comparison of means. The chi-square-test was used for comparisons of prevalence between groups. The associations between the dependent variable (SF-36 subscales) and independent variables (the suggested health factors; feeling painless, sleep structure, feeling rested, diet, exercise, performing hobbies, feeling sexual lust, SOC, social support, alcohol habit, immigrant status, civil status, education, work capacity, socioeconomic status - main occupation, socioeconomic status - current occupation, and rheumatic disease) were estimated by odds ratios (OR) and 95% confidence intervals (CI) calculated by multivariable logistic regressions for each of the variables at a time with adjustment for sex, age and baseline SF-36 values. The analyses were done with simple contrast to a reference group for each of the independent variables. Individuals with missing values for any of the variables were rejected from the analyses. The actual number of individuals in each analysis is reported in tables 2 and 3, and was considered to fulfil the requirement of at least 10 individuals in the outcome for each independent variable. A *p*-value of less than 0.05 was considered statistically significant. A power calculation showed that at least 150 individuals would be enough. The power calculation was based on the analysis of the SF-36 vitality scale and a power of more than 80% for a two-tailed test, a significance level of 5% and an assumption that the minimum difference between the groups was 6 points and the maximum standard deviation was 20 points [33].

**Table 2:** Proposed health factors at baseline, and outcome in HRQL at the 12 month follow-up.

		PF	RP	BP	GH	VT	SF	RE	MH
		n = 185 OR (95% CI)	n = 185 OR (95% CI)	n = 185 OR (95% CI)	n = 185 OR (95% CI)	n = 185 OR (95% CI)	n = 185 OR (95% CI)	n = 185 OR (95% CI)	n = 185 OR (95% CI)
Sex	Women	1.0	1.0	1.0	1.0	1.0	1.0	1.0	1.0
	Men	1.0 (0.4-2.3)	1.2 (0.6-2.6)	1.3 (0.6-2.6)	1.7 (0.8-3.8)	0.7 (0.4-1.5)	1.6 (0.8-3.4)	1.4 (0.6-3.0)	1.1 (0.5-2.5)
									
Age	70-88	1.0	1.0	1.0	1.0	1.0	1.0	1.0	1.0
(years)	61-69	3.1 (0.9-9.6)	1.1 (0.4-3.2)	1.1 (0.5-2.8)	0.8 (0.3-2.2)	0.8 (0.3-1.9)	0.8 (0.3-2.0)	2.1 (0.8-5.4)	1.8 (0.7-4.9)
	52-60	3.8 (1.2-11.8)	3.3 (1.2-9.0)	1.7 (0.7-4.1)	1.3 (0.5-3.4)	1.1 (0.4-2.6)	1.4 (0.6-3.5)	6.4 (2.3-17.3)	2.3 (0.9-6.3)
	18-51	3.6 (1.2-11.2)	1.8 (0.7-4.9)	0.8 (0.3-1.9)	0.9 (0.3-2.3)	0.6 (0.3-1.5)	0.9 (0.4-2.2)	2.6 (1.0-6.4)	1.7 (0.7-4.4)
									
SF-36 at	≥mean	1.0	1.0	1.0	1.0	1.0	1.0	1.0	1.0
baseline	<mean	24.7 (10.3-59.2)	5.6 (2.8-11.3)	4.4 (2.2-8.8)	9.6 (4.8-19.2)	4.5 (2.4-8.7)	5.4 (2.8-10.3)	7.8 (3.6-16.6)	10.7 (5.2-22.0)
									
Feeling painless	8-10	1.0	1.0	1.0	1.0	1.0	1.0	1.0	1.0
	6-7	0.4 (0.1-1.8)	1.4 (0.4-5.3)	2.5 (0.7-8.7)	0.6 (0.2-1.9)	1.4 (0.4-4.3)	1.2 (0.4-3.7)	0.9 (0.3-2.9)	2.6 (0.7-9.2)
	4-5	0.3 (0.1-1.4)	1.4 (0.4-5.5)	5.5 (1.6-19.3)	0.7 (0.2-2.3)	1.7 (0.5-5.6)	3.0 (0.9-9.7)	0.8 (0.3-2.7)	1.7 (0.5-6.4)
	0-3 Nearly painless	0.4 (0.1-1.8)	2.2 (0.6-8.4)	7.9 (1.8-35.3)	1.1 (0.3-3.5)	2.9 (0.9-9.4)	2.1 (0.6-6.8)	1.4 (0.4-4.7)	2.2 (0.6-8.0)
									
Sleep structure	Big/very big problem	1.0	1.0	1.0	1.0	1.0	1.0	1.0	1.0
	Moderate problem	1.6 (0.6-3.8)	1.3 (0.6-2.9)	2.0 (0.9-4.2)	2.1 (0.9-4.7)	2.1 (1.0-4.4)	2.0 (0.9-4.3)	1.4 (0.7-3.1)	1.2 (0.5-2.6)
	No/small problem	1.1 (0.4-3.1)	1.9 (0.8-4.7)	1.7 (0.7-4.1)	3.1 (1.2-7.8)	3.3 (1.4-7.8)	1.9 (0.8-4.7)	1.0 (0.4-2.3)	0.9 (0.4-2.3)
									
Feeling rested	Big/very big problem	1.0	1.0	1.0	1.0	1.0	1.0	1.0	1.0
	Moderate problem	1.2 (0.5-3.2)	2.2 (0.9-5.3)	1.9 (0.9-4.2)	1.6 (0.7-3.8)	3.2 (1.4-7.1)	1.4 (0.6-3.1)	1.6 (0.7-3.6)	2.0 (0.8-4.6)
	No/small problem	1.2 (0.5-3.1)	2.6 (1.0-6.4)	4.0 (1.7-9.3)	2.5 (1.0-5.8)	4.6 (2.0-10.6)	3.4 (1.4-8.2)	1.5 (0.7-3.5)	1.5 (0.6-3.6)
									
Diet	General diet	1.0	1.0	1.0	1.0	1.0	1.0	1.0	1.0
	Special diet	1.4 (0.5-4.6)	1.1 (0.4-3.0)	0.8 (0.3-1.9)	0.6 (0.2-1.7)	1.6 (0.6-4.3)	0.7 (0.3-1.9)	0.9 (0.3-2.3)	2.1 (0.7-6.2)
									
High effort	Irregularly/never	1.0	1.0	1.0	1.0	1.0	1.0	1.0	1.0
of exercise	1 time a week	1.0 (0.3-3.0)	1.0 (0.4-2.7)	0.9 (0.4-2.3)	1.2 (0.4-3.3)	1.1 (0.4-2.8)	0.4 (0.1-0.9)	1.2 (0.4-3.3)	0.4 (0.1-1.1)
	>2 times a week	0.9 ( 0.4-2.3)	1.4 (0.6-3.1)	1.1 (0.5-2.4)	1.4 (0.6-3.2)	1.4 (0.7-3.0)	0.5 (0.3-1.2)	1.0 (0.5-2.3)	0.5 (0.2-1.1)
									
Medium effort	Irregularly/never	1.0	1.0	1.0	1.0	1.0	1.0	1.0	1.0
of exercise	1 time a week	2.1 (0.6-7.7)	0.4 (0.1-1.6)	0.5 (0.2-1.4)	0.7 (0.2-2.3)	0.9 (0.3-2.7)	0.3 (0.1-0.9)	0.9 (0.3-2.9)	0.6 (0.2-2.0)
	>2 times a week	2.0 (0.8-4.6)	1.3 (0.6-2.8)	1.6 (0.8-3.2)	1.9 (0.9-4.1)	1.3 (0.6-2.6)	0.8 (0.4-1.6)	1.0 (0.5-2.0)	1.3 (0.6-2.8)
									
Low effort	Irregularly/never	1.0	1.0	1.0	1.0	1.0	1.0	1.0	1.0
of exercise	1 time a week	1.4 (0.3-6.5)	0.6 (0.2-2.6)	0.7 (0.2-2.3)	0.9 (0.2-3.4)	1.0 (0.3-3.6)	0.8 (0.2-3.0)	1.3 (0.3-4.7)	1.6 (0.4-6.2)
	>2 times a week	2.8 (1.1-7.3)	1.4 (0.6-3.3)	1.1 (0.6-2.3)	1.4 (0.6-3.0)	1.4 (0.7-2.8)	1.6 (0.8-3.4)	1.8 (0.8-3.9)	1.5 (0.7-3.3)
									
Performing hobbies	Rarely/never	1.0	1.0	1.0	1.0	1.0	1.0	1.0	1.0
	Often/sometimes	1.5 (0.6-3.5)	1.7 (0.8-3.7)	0.8 (0.4-1.7)	1.1 (0.5-2.4)	0.9 (0.5-1.8)	0.9 (0.5-1.8)	1.1 (0.6-2.3)	0.9 (0.4-1.9)
									
Feeling sexual lust	Rarely/never	1.0	1.0	1.0	1.0	1.0	1.0	1.0	1.0
	Often/sometimes	1.5 (0.7-3.5)	1.0 (0.5-2.1)	0.7 (0.4-1.4)	0.5 (0.3-1.2)	0.8 (0.4-1.6)	0.6 (0.3-1.3)	1.8 (0.9-3.8)	2.0 (0.9-4.4)

OR (95% CI) in multivariate analyses of factors believed to affect HRQL (assessed by SF-36) in a positive way in a population with rheumatic diseases at a 12 month follow-up. Factors were controlled for age, sex and baseline SF-36 score but not for each other.

**Table 3:** Proposed health factors at baseline, and outcome in HRQL at the 12 month follow-up.

		PF	RP	BP	GH	VT	SF	RE	MH
		n = 185 OR (95% CI)	n = 185 OR (95% CI)	n = 185 OR (95% CI)	n = 185 OR (95% CI)	n = 185 OR (95% CI)	n = 185 OR (95% CI)	n = 185 OR (95% CI)	n = 185 OR (95% CI)
SOC	21-55	1.0	1.0	1.0	1.0	1.0	1.0	1.0	1.0
	56-66	1.2 (0.4-3.4)	2.9 (0.9-8.5)	3.0 (1.2-7.2)	3.1 (1.1-8.4)	2.8 (1.1-7.0)	2.4 (0.9-5.8)	6.3 (2.3-17.7)	4.7 (1.7-12.7)
	67-75	1.7 (0.5-5.1)	3.4 (1.2-9.8)	3.3 (1.3-8.1)	3.1 (1.1-8.6)	2.7 (1.1-7.0)	3.2 (1.2-8.2)	5.6 (2.0-15.5)	7.5 (2.5-22.4)
	75-90 Very good	1.6 (0.5-5.2)	4.9 (1.6-15.6)	5.6 (2.0-15.5)	5.1 (1.7-15.8)	7.8 (2.6-23.4)	9.0 (2.9-27.9)	11.8 (3.5-39.7)	10.1 (2.9-35.1)
									
Social support	18-29	1.0	1.0	1.0	1.0	1.0	1.0	1.0	1.0
	13-17	2.9 (0.9-9.1)	1.6 (0.5-4.4)	1.6 (0.6-4.0)	2.2 (0.8-6.2)	1.2 (0.5-3.2)	1.5 (0.6-3.9)	3.0 (1.1-8.1)	1.3 (0.5-3.5)
	11-12	2.2 (0.6-7.2)	1.9 (0.7-5.7)	1.2 (0.5-3.3)	1.6 (0.6-4.9)	1.9 (0.7-5.3)	1.3 (0.5-3.6)	6.0 (1.9-18.5)	2.5 (0.8-7.5)
	10 Very good	2.2 (0.7-6.9)	2.2 (0.8-6.3)	1.8 (0.7-4.6)	2.4 (0.9-6.6)	2.1 (0.8-5.4)	1.8 (0.7-4.6)	5.2 (1.8-15.0)	1.6 (0.6-4.4)
									
Alcohol habit	Rarely/never	1.0	1.0	1. 0	1.0	1.0	1.0	1.0	1.0
	Monthly	1.1 (0.4-2.9)	0.7 (0.3-1.7)	0.8 (0.4-1.8)	1.1 (0.5-2.5)	0.7 (0.3-1.5)	1.1 (0.5-2.5)	0.9 (0.4-2.2)	1.4 (0.6-3.3)
	Weekly	1.0 (0.4-2.5)	0.6 (0.3-1.5)	1.0 (0.5-2.2)	0.9 (0.4-2.0)	1.5 (0.7-3.2)	1.2 (0.5-2.6)	0.8 (0.4-1.8)	0.6 (0.3-1.5)
									
Immigrant status	Immigrant	1.0	1.0	1.0	1.0	1.0	1.0	1.0	1.0
	Swede	0.7 (0.2-2.6)	1.5 (0.5-4.4)	2.2 (0.8-5.8)	0.9 (0.3-2.4)	0.6 (0.2-1.6)	2.1 (0.8-5.5)	1.5 (0.5-4.2)	2.8 (0.9-8.0)
									
Civil status	Living alone	1.0	1.0	1.0	1.0	1.0	1.0	1.0	1.0
	Living with	0.9 (0.4-2.2)	0.7 (0.3-1.4)	0.6 (0.3-1.2)	1.0 (0.5-2.1)	0.5 (0.3-1.1)	0.6 (0.3-1.3)	1.0 (0.5-2.1)	0.6 (0.3-1.2)
	somebody								
									
Education	Grade school	1.0	1.0	1.0	1.0	1.0	1.0	1.0	1.0
	Secondary school	1.4 (0.6-3.6)	1.3 (0.6-3.0)	1.5 (0.7-3.2)	1.3 (0.6-2.9)	1.0 (0.5-2.2)	3.1 (1.4-7.0)	1.2 (0.5-2.6)	1.9 (0.8-4.3)
	College/university	2.1 (0.8-5.7)	1.7 (0.7-4.0)	1.2 (0.6-2.7)	0.8 (0.3-1.8)	1.4 (0.6-3.0)	1.6 (0.7-3.6)	1.4 (0.6-3.1)	1.7 (0.7-4.0)
									
Work capacity	0%	1.0	1.0	1.0	1.0	1.0	1.0	1.0	1.0
	25-100%	3.3 (1.2-9.2)	3.0 (1.2-7.2)	2.7 (1.2-6.0)	1.9 (0.8-4.7)	1.6 (0.7-3.6)	1.7 (0.8-4.0)	1.8 (0.8-4.4)	4.3 (1.6-11.5)
	Retired	0.9 (0.2-4.6)	3.7 (0.6-21.6)	1.6 (0.4-6.3)	2.7 (0.6-11.3)	1.4 (0.4-5.4)	1.2 (0.3-4.8)	0.8 (0.2-3.2)	0.8 (0.2-3.9)
									
Socioeconomic	Group A	1.0	1.0	1.0	1.0	1.0	1.0	1.0	1.0
status,	Group B	1.5 (0.6-4.3)	2.0 (0.8-5.1)	1.2 (0.5-2.7)	0.5 (0.2-1.2)	1.4 (0.6-3.2)	1.7 (0.7-4.0)	1.8 (0.7-4.2)	1.5 (0.6-3.8)
main	Group C	1.5 (0.6-4.0)	1.6 (0.6-3.8)	0.9 (0.4-2.0)	0.8 (0.4-1.9)	1.1 (0.5-2.4)	1.4 (0.6-3.1)	2.2 (0.9-5.2)	1.6 (0.7-3.9)
occupation	Group D	0.3 (0.1-1.8)	0.4 (0.1-2.2)	0.5 (0.1-2.0)	0.4 (0.1-2.0)	1.9 (0.5-7.3)	1.3 (0.3-5.1)	1.1 (0.3-4.5)	2.3 (0.5-9.8)
									
Socioeconomic	Group A	1.0	1.0	1.0	1.0	1.0	1.0	1.0	1.0
status,	Group B	0.7 (0.1-4.4)	3.3 (0.6-17.9)	1.4 (0.3-6.6)	0.7 (0.1-4.2)	1.1 (0.3-5.0)	3.3 (0.6-17.9)	1.8 (0.3-10.0)	4.6 (0.6-35.7)
current	Group C	0.9 (0.2-3.7)	2.9 (0.8-11.1)	1.0 (0.3-3.2)	0.4 (0.1-1.4)	1.2 (0.4-3.8)	1.8 (0.6-6.1)	1.9 (0.5-7.8)	3.9 (0.9-16.7)
occupation	Group D	0.4 (0.1-1.5)	1.5 (0.4-5.4)	0.3 (0.1-0.9)	0.1 (0.0-0.5)	0.5 (0.2-1.3)	0.9 (0.3-2.6)	0.6 (0.2-1.9)	0.7 (0.2-2.3)
									
Rheumatic	Local/general pain	1.0	1.0	1.0	1.0	1.0	1.0	1.0	1.0
disease	Osteoarthritis	2.2 (0.5-9.8)	1.4 (0.3-5.8)	0.7 (0.2-2.3)	2.4 (0.6-9.3)	1.3 (0.4-4.7)	1.2 (0.3-4.1)	0.7 (0.2-2.7)	1.1 (0.3-4.1)
	Systemic rheumatic disease	0.9 (0.2-4.5)	0.8 (0.2-3.7)	0.9 (0.3-3.1)	0.6 (0.1-3.1)	0.9 (0.2-3.5)	1.0 (0.3-3.7)	0.6 (0.2-2.3)	1.1 (0.3-4.4)
	Infl. joint disease	2.1 (0.7-6.2)	2.1 (0.7-6.2)	1.1 (0.4-2.8)	2.0 (0.7-5.7)	2.5 (0.9-6.8)	1.8 (0.7-4.7)	0.6 (0.2-1.6)	2.0 (0.7-5.6)

OR (95% CI) in multivariate analyses of factors believed to affect HRQL (assessed by SF-36) in a positive way in a population with rheumatic diseases at a 12 month follow-up. Factors were controlled for age, sex and baseline SF-36 score but not for each other.

Group A: Manual workers

Group B: Assistant no manual employees

Group C: Intermediate/higher employees and upper-level executives

Group D: Others

### Ethics

The study was approved by the Ethics Research Committee, Faculty of Medicine, Lund University, Sweden, dnr 566/2006.

## Results

**Figure 1:**
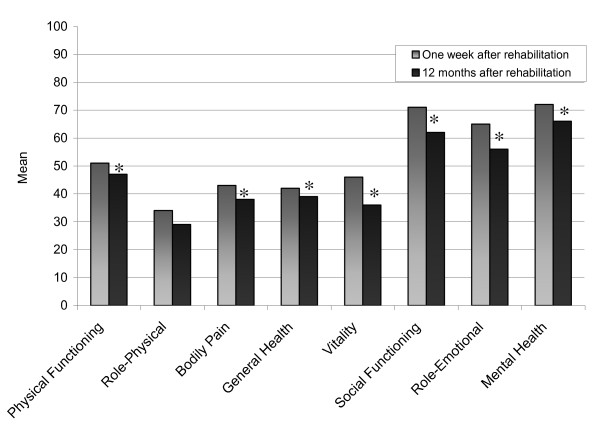
**The SF-36 scores for people with rheumatic diseases**. Comparison of the SF-36 subscales scores (mean values) for people with rheumatic diseases at baseline one week after rehabilitation and at the 12 month follow-up. * = Significant change (*p *< 0.05)

The most common group of rheumatic diseases was inflammatory joint diseases (63%). There was a predominance of women (75%) (Table 1), and the mean age was 59.4 years. There were significant deteriorations in seven of the SF-36 dimensions between the one week and the 12 month follow-up. The mean changes were for PF 3.9 points (*p *= 0.004), RP 5.2 points (*p *= 0.080), BP 5.1 points (*p *= 0.002), GH 2.7 points (*p *= 0.016), VT 9.5 points (*p *= < 0.001), SF 8.5 points (*p *= < 0.001), RE 9.7 points (*p *= 0.006) and MH 5.1 points (*p *= 0.001) (Figure 1).

### Factors impact on HRQL at the 12 month follow-up

The predictive value of the suggested health factors with regard to HRQL over 12 months was estimated with multivariable logistic regression analyses, where each of the health factors was controlled for sex, age and baseline SF-36. Results from the multivariable logistic regressions with OR and 95% CI for these variables are found in tables 2 and 3.

*Sex *was not associated with having a health status better than the mean score in any of the SF-36 subscales at the 12 month follow-up. Younger *age *(18-51 years) significantly (*p *< 0.05) predicted a health status better than the mean score in PF and RE compared to older age (70-88 years). Being 52-60 years significantly predicted a better outcome in PF, RP and RE compared to older age (70-88 years).

A lower score (indicating less pain) in *feeling painless *significantly (*p *< 0.05) predicted a health status better than the mean score in BP at the 12 month follow-up, compared to feeling strong pain (8-10 points). Having no/small problem with the *sleep structure *predicted a better outcome in GH and VT, and having moderate problem with the sleep structure predicted a better outcome in VT compared to big/very big problem with the sleep structure. *Feeling rested *after sleep predicted a better outcome in five of the SF-36 subscales, RP, BP, GH, VT and SF, compared to reporting big/very big problem with not feeling rested. Moderate problem with feeling rested after sleep predicted a better outcome in VT compared to reporting big/very big problem with not feeling rested.

The special *diet *was not associated with having a health status better than the mean score in any of the SF-36 subscales at the 12 month follow-up compared to general diet. *High *or *medium effort of exercise *once per week predicted a worse outcome in SF compared to do irregularly/never effort of exercise. *Low effort of exercise *more than twice per week predicted a better outcome in PF compared to do irregularly/never effort of exercise. *Performing hobbies *or *feeling sexual lust *often/sometimes was not associated with having a health status better than the mean score in any of the SF-36 subscales at the 12 month follow-up compared to performing hobbies or feeling sexual lust rarely/never.

A strong/very good or a slightly weaker *SOC *significantly (*p *< 0.05) predicted a health status better than the mean score in seven of the SF-36 subscales, RP, BP, GH, VT, SF, RE and MH, at the 12 month follow-up compared to a very weak SOC (21-55 points). A weaker SOC predicted a better outcome in BP, GH, VT, RE and MH compared to a very weak score. All lower and better scores of *social support *(emotional support and practical assistance) predicted a better outcome in RE compared to the highest and worst score (18-29 points).

The *alcohol habit *(weekly compared to rarely/never drink alcohol)*, immigrant status *(Swede compared to immigrant) or *civil status *(living with somebody compared with living alone) were not associated with having a health status better than the mean score in any of the SF-36 subscales at the 12 month follow-up. Secondary school as highest *education *predicted a better outcome in SF compared to grade school.

A *work capacity *of 25-100% significantly (*p *< 0.05) predicted a health status better than the mean score in PF, RP, BP and MH at the 12 month follow-up compared to 0% work capacity. The *socioeconomic status, main occupation *(assistant no manual employees, intermediate/higher employees and upper-level executives, and others) did not predict any better outcome in SF-36 compared to manual workers. The *socioeconomic status, current occupation *and the group D (Others) predicted a worse outcome in BP and GH compared to manual workers. *The rheumatic disease *(inflammatory joint disease, systemic rheumatic disease and osteoarthritis) was not associated with having a health status better than the mean score in any of the SF-36 subscales at the 12 month follow-up compared to local/general pain.

The multivariate logistic regression analyses were not intended to be complete explanatory models, but at the 12 month follow-up 48.8-55.3% of the variance in PF could be explained by the predictor variables (Nagelkerke R^2^) and 21.2-33.5% in RP, 14.3-25.6% in BP, 31.1-43.9% in GH, 16.6-26.0% in VT, 20.3-30.1% in SF, 28.0-40.1% in RE and 32.3-44.0% in MH.

## Discussion

The focus in this study was on the effect of proposed health factors on the long-term outcome in HRQL in people with rheumatic diseases that had the same starting point, they had undergone inpatient rehabilitation. The individuals had a statistically significant deterioration in SF-36 between baseline at one week and the follow-up 12 months after the rehabilitation. The health factors that proved to affect most subscales in HRQL in a positive way were having a strong SOC, feeling rested after sleep, having work capacity, being younger or middle-aged, and having no/small problem with the sleep structure.

The most obvious health factor was having strong SOC which was predicting a positive outcome in seven of the eight SF-36 subscales at the 12 month follow-up. This agreed well with the results from Abu-Shakra et al. where SOC had a major influence on the quality of life in individuals with SLE. These individuals with strong SOC had the ability to predict, explain and cope with disease related stressors and achieve a better quality of life [13]. The same results were shown in individuals with scleroderma [34]. Antonovsky meant that the SOC changed very little in adulthood, if the individual was not exposed to major changes in life like moving to another place, giving birth to a child or going into rehabilitation [10]. It has been shown that SOC is only stable for individuals with initially high scores of SOC. For individuals with lower SOC, the condition of disease and societal changes influences the score [35]. This indicates that the SOC can be affected. SOC is therefore an important domain to take into account and to study more in order to develop treatment strategies that could help health-care professionals to strengthen the HRQL in individuals with rheumatic diseases.

Another important health factor in the present study was feeling rested after sleep. The same result was shown in an eight-year follow-up of individuals with or without chronic musculoskeletal pain [36]. Some of the main factors that influence the quality of life negatively in individuals with RA are sleep disturbance and fatigue [37]. Fatigue is a common problem in individuals with chronic illnesses and this subjective symptom worries the individuals. Healthcare professionals have to learn communication techniques to help individuals to express concerns about fatigue [38,39]. More research is needed to find the best way of treating fatigue in people with rheumatic diseases.

Having work capacity was also an important health factor. This agreed well with another study where external factors like employment and having financial support were important to secure health. External factors were strongly linked to the perceptions of normality [40]. There is an association between work disability and RA. Factors that contribute to decisions to cease work are the physical nature of the work, the workload, the fatigue or the pain [41]. Conversely, the present study showed that the work capacity contributed to a higher score in health status. It is therefore important to help individuals to be able to continue to work, regardless of the number of working hours, but adjustments of the nature of the work and workload have to be made.

Age was also an important health factor in this study. Being of younger age (18-51 years) or middle-age (52-60 years) was associated with a better health status. The same pattern has been reported in individuals with or without chronic musculoskeletal pain; however, the significant ages were younger (20-33 years and 34-46 years) [36] compared with the present study.

Another important health factor was having no/small problem with the sleep structure. This also agreed well with the eight-year follow-up of individuals with or without chronic musculoskeletal pain [36]. The conclusion of a review was that a good sleep is vital for the health and HRQL. However, the role of sleep is unfortunately not well explored [42]. These results indicate that a good sleep structure is an important domain to work within clinical practice. More research must be done on how to influence towards a better sleep structure for people with rheumatic diseases.

Other health factors were feeling painless, making low effort exercise more than two times per week, having emotional support and practical assistance, and having completed secondary school as highest education. Each of these factors predicted a better health status in only one of the SF-36 subscales. High or medium effort of exercise predicted a worse health status in one of the SF-36 subscales. Other studies have shown the importance of these factors on the health status. It is well known that pain [20,43] and a low education level [3,31] are risk factors that could decrease HRQL, and that having emotional support could increase HRQL [31,36]. The effect of exercise on HRQL is more complex, and there are no consistent results [7,8,14,15]. There is nevertheless more evidence that exercise is beneficial than risky [15].

It was surprising that the diet, performing hobbies, feeling sexual lust, alcohol habit, immigrant status, civil status, socioeconomic status - main occupation, socioeconomic status - current occupation and rheumatic disease were not affecting any subscale in the health status. In clinical practice patients often mention these factors as very important for their health status.

In the eight SF-36 subscales the mean changes were only deteriorations of 2.7-9.7 points after 12 months and if these few points are of clinical significance is controversial. Valuation of the clinical relevance of mean changes in SF-36 is ongoing [44] but there is a suggestion that effects larger than 12% of the baseline value in SF-36 are assumed to be the minimal clinically important differences (MCID) [45]. In the present study there were >12% deterioration in the subscale RP, BP, VT, SF and RE. This could prove that the deterioration had a clinical significance for the individuals HRQL.

All the individuals in the present study had rheumatic diseases and had completed a multimodal rehabilitation when the study started. However, the aim was not to evaluate the rehabilitation but rather to find factors predicting a better health status 12 months after the rehabilitation in people with rheumatic diseases. It has been impossible to determine if any of these health factors were interacting with the rehabilitation program since there was no control group.

Finally, future longitudinal studies comparing health promoting factors are needed to confirm their impact on HRQL in people with rheumatic diseases. There is also a need for more studies about how individuals' SOC could be strengthened and if health-care professionals could help them with that. Health factors as well as risk factors are important to address in clinical work. Strategies have to be formed to help people with rheumatic diseases to identify and strengthen factors like feeling rested after sleep, having a good sleep structure and having work capacity to improve their health status.

### Methodological considerations

The number of individuals in the study did not allow for full multivariate models including all independent variables, so it was decided to introduce the variables in separate analyses, controlling for age, sex and baseline value of the SF-36 subscales. In lack of any known valid cut-point for good health in this population, when dichotomising the SF-36 subscales it was decided to use the mean value for each of the subscales as a cut-point, in order to get enough individuals in each group of the dependent variable.

As age and sex were likely to be confounders, these factors were controlled for in the analyses, together with the baseline values of the SF-36 subscales, to adjust for the possibility that outcome would reflect the baseline score and not a change over time.

There is a problem in the use of SF-36 that floor and roof effects can reduce the possible change over time in the extreme ends of the scales.

A limitation in this study was that a *p*-value of less than 0.05 was considered statistically significant; however, because of the many comparisons *p*-values showing a weak significance (>0.01) may appear by chance.

## Conclusions

The most important health factors were having a strong SOC, feeling rested after sleep, having work capacity, being younger or middle-aged, and having no/small problem with the sleep structure. These health factors are important to put forward and address in clinical work with rheumatic diseases. Knowledge of factors predicting a good health outcome should be used to optimise treatment strategies.

## Competing interests

The authors declare that they have no competing interests.

## Authors' contributions

All authors contributed equally in designing the study, discussing the statistical framework, interpretation and discussion of the findings. SA and SB carried out the statistical analyses and drafted the manuscript. All authors read and approved the final manuscript.

## Pre-publication history

The pre-publication history for this paper can be accessed here:

http://www.biomedcentral.com/1471-2474/12/102/prepub
